# Varying influences of selection and demography in host-adapted populations of the tick-transmitted bacterium, *Anaplasma phagocytophilum*

**DOI:** 10.1186/s12862-015-0335-z

**Published:** 2015-03-31

**Authors:** Matthew L Aardema, Friederike D von Loewenich

**Affiliations:** Department of Ecology and Evolutionary Biology, Princeton University, Princeton, NJ USA; Department of Medical Microbiology and Hygiene, University of Mainz, Medical Centre, Mainz, Germany

**Keywords:** *Anaplasma phagocytophilum*, Bacteria, Zoonoses, Host range, Effective population size, Demography, Selection

## Abstract

**Background:**

The host range of a pathogenic bacterial strain likely influences its effective population size, which in turn affects the efficacy of selection. Transmission between competent hosts may occur more frequently for host generalists than for specialists. This could allow higher bacterial population densities to persist within an ecological community and increase the efficacy of selection in these populations. Conversely, specialist strains may be better adapted to their hosts and consequently achieve greater within-host population densities, with corresponding increases in selection efficacy. To assess these different hypotheses, we examined the effective population sizes of three strains of the bacterium *Anaplasma phagocytophilum* and categorized the varying roles of selection and demography on patterns of genetic diversity and divergence in these populations. *A. phagocytophilum* is a tick-transmitted, obligately intracellular pathogen. Strains of *A. phagocytophilum* display varying degrees of host specialization, making this a good species for exploring questions regarding host range, effective population size and selection efficacy.

**Results:**

We found that a roe deer specialist harbored the most genetic diversity of the three *A. phagocytophilum* strains and correspondingly had the largest effective population size. Another strain that is ecologically specialized on rodents and insectivores had the smallest effective population size. However, these mammalian hosts are distantly related evolutionarily. The third strain, a host generalist, was intermediate in its effective population size between the other two strains. Evolutionary constraint on non-synonymous sites was pervasive in all three strains, although some slightly deleterious mutations may also be segregating in these populations. We additionally found evidence of genome-wide selective sweeps in the generalist strain, whereas signals of repeated bottlenecks were detected in the strain with the smallest effective population size.

**Conclusions:**

*A. phagocytophilum* is a diverse bacterial species that differs among distinct strains in its effective population size, as well as how genetic diversity and divergence have been influenced by selection and demographic changes. In this species, host specialization may facilitate increased population growth and allow more opportunities for selection to act. These results provide insights into how host range has influenced evolutionary patterns of strain divergence in an emerging zoonotic bacterium.

**Electronic supplementary material:**

The online version of this article (doi:10.1186/s12862-015-0335-z) contains supplementary material, which is available to authorized users.

## Background

Obligately intracellular bacteria typically have smaller effective population sizes than free-living relatives due to constraints placed on them by the cellular space needed for growth, the number of cells capable of being infected and the availability of competent hosts [1]. Among pathogenic bacteria, variation in host range may also influence effective population sizes as the diversity of competent host species available for infection influences both transmission dynamics and disease prevalence in the environment [2,3]. Population densities, connectivity and immune responses can also vary among the different species a pathogen is capable of infecting [4-6]. This may further impact effective population sizes and the levels of genetic diversity observed between strains.

Variation in host-range may also influence intra-specific strain divergence. Small population sizes and limited transmission between hosts can result in strong genetic bottlenecks, which reduce diversity and create the potential for genetic divergence to arise between strains through drift [7-9]. Adaptation to different hosts could also be an important contributor of strain divergence [1]. In addition to producing divergence at the target of selection, such adaptive evolution is often accompanied by a selective sweep, which may create additional genetic divergence between strains [10]. The relative roles of stochastic evolutionary processes and directed, adaptive evolution have not been well categorized in pathogenic bacterial populations, but both are known to make important contributions to evolution in free-living bacteria and other clonal organisms [10-12].

**Figure 1:**
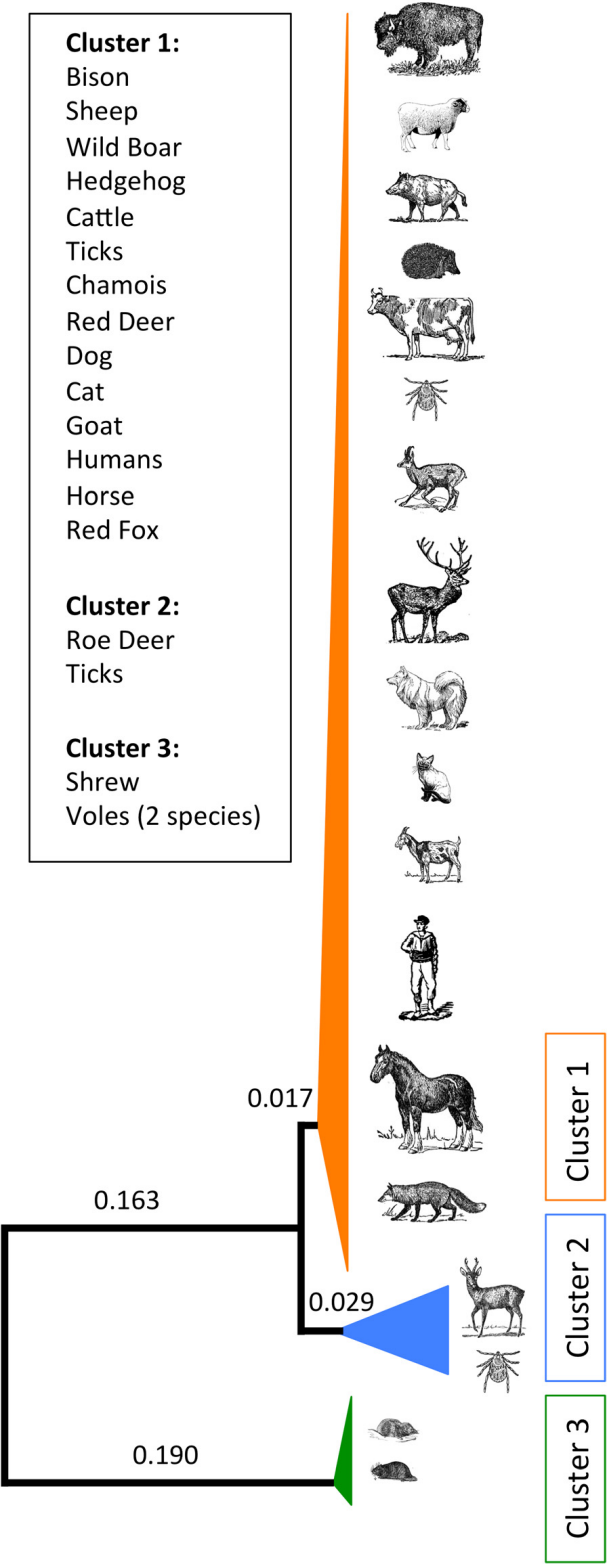
**Phylogeny of the three** ***A. phagocytophilum*** **strains used in this study based on seven conserved genetic regions.** Rather than display each branch tip individually we have chosen to represent each of the three clusters with a composite triangle. The vertical height of the triangle indicates relative sample sizes for each strain examined in this study. The horizontal width of the triangle indicates the extent of intra*-*strain synonymous genetic diversity. The numbers along each branch indicate the observed number of synonymous substitutions per synonymous site (d_S_). The vertical order of the taxa displayed within each cluster is arbitrary.

To examine the influence of host range variation on effective population size and intra-specific evolutionary divergence, we examined genetic diversity among distinct populations of the obligately intercellular bacterium *Anaplasma phagocytophilum*. As a bacterial species, this emerging zoonotic pathogen infects a broad range of vertebrate hosts [4]. However, there are multiple, discrete strains of *A. phagocytophilum* circulating in Europe, with minimal overlap in their host associations [13-15]. One of these strains is a generalist with a relatively broad host range, encompassing mammal species from a wide taxonomic spectrum (Figure 1). This is also the strain that overwhelmingly infects humans, livestock and other domestic animals [13-15]. By contrast, a second strain appears to specialize almost exclusively on roe deer. Both of these strains share the same primary tick vector, *Ixodes ricinus*. A third strain of mammal-infecting *A. phagocytophilum* is also circulating in Europe, but it is unclear whether this population can be classified as either a specialist or generalist. It predominately infects small mammals such as voles and shrews. This strain’s ecologically narrow host range is due in part to transmission by the nest-living tick species *Ixodes trianguliceps* [13]. However, while its potential hosts may share many ecological similarities, rodents and insectivores are among the most evolutionary divergent mammals capable of harboring *A. phagocytophilum*. Each belongs to a distinct superorder and their respective lineages diverged between 74 and 98 million years ago [16]. Immunity and other factors affecting bacterial population growth may differ greatly between these hosts [17], which likely constrains the ability of *A. phagocytophilum* to adapt to these species.

In this study we compare two distinct hypotheses related to host range in *A. phagocytophilum*. The first hypothesis is that generalist strains will maintain larger effective population sizes than specialist strains. As generalists should have a higher density of hosts to colonize within an ecological community, greater transmission potential between hosts should be higher leading to increased population sizes. Alternatively, specialists may achieve higher within-host population densities due to increased adaptation to their hosts. This could lead to overall higher effective population sizes relative to generalists that are more poorly adapted to any particular host species. In conjunction with these predicted differences between host specialists and generalists, we also postulated that the relative importance of selection and drift in producing evolutionary divergence between strains differs in relation to effective population size.

To test our hypotheses we examined genetic data from 265 individual *A. phagocytophilum* samples obtained from 17 European mammal species and *Ixodes ricinus* ticks. These samples cluster into the three distinct strains described above. With this data we examined the amount of standing genetic diversity harbored in each strain and estimated their effective population sizes. We also explored the contributions of selection and drift to the production of divergence between these populations.

## Results

### Genetic diversity and effective population sizes

Following Huhn and colleagues [14], we will refer to the generalist strain of *A. phagocytophilum* as ‘cluster 1’ and the roe deer specialist strain as ‘cluster 2’ (Figure 1). We will refer to the population that infects voles and shrews and is transmitted by a distinct tick vector as the ‘cluster 3’ strain. Using population data from partial sequences of seven housekeeping genes, we estimated two measures of genetic diversity for each strain. The first measure was π, which is the mean pairwise genetic difference between samples [18,19]. The second was θ_W,_ which is a measure of genetic diversity based on the number of segregating mutations in a sample [20].

**Figure 2:**
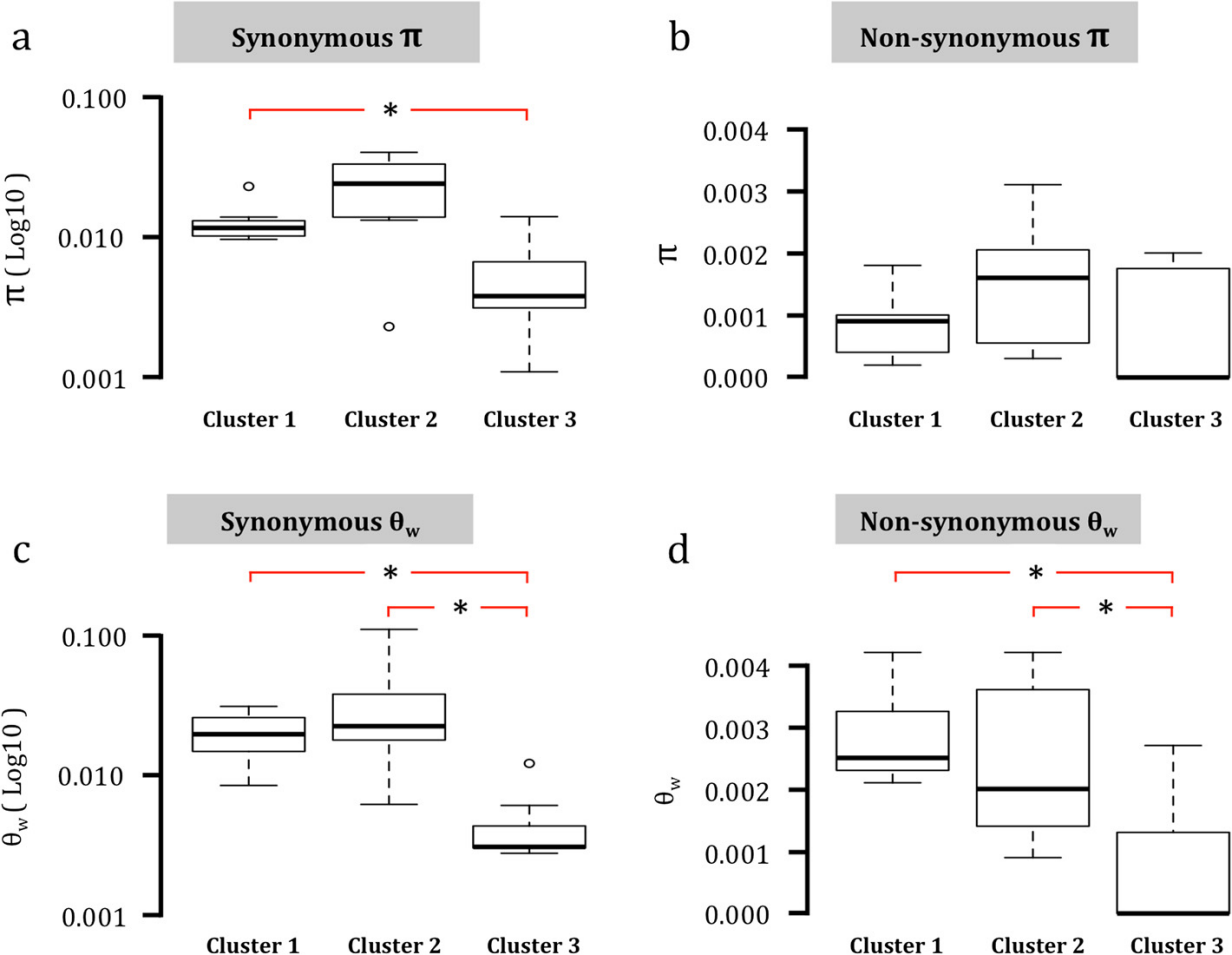
**Boxplots showing the median values (thick horizontal black lines) and quartiles for π (a & b) and θ**_**w**_ **(c & d) estimates at synonymous and non-synonymous sites.** Open dots indicate outlying estimates. The red lines with asterisks indicate significant differences between clusters based on a paired t-test (p < 0.05). Note that for both synonymous π and θ_w_ the y-axis is on a Log10 scale.

In agreement with our second hypothesis, we found that on average the cluster 2 strain harbored more pairwise genetic diversity than either clusters 1 or 3 (Figure 2a-d, Table 1, Additional file 1: Table S1). For non-synonymous π estimates, differences between strains were not significant (paired t tests, 1 vs. 2: t_6_ = -1.640, p = 0.152; 1 vs. 3: t_6_ = 0.057, p = 0.956; 2 vs. 3: t_6_ = 1.240, p = 0.261). For synonymous diversity, cluster 1 was significantly different from cluster 3 (paired t test, t_6_ = 3.074, p = 0.022), but despite having a higher average diversity level, cluster 2 was not significantly different from cluster 3 (paired t test, t_6_ = 1.928, p = 0.102). This is likely due to the high amount of variance in π estimates observed between loci in cluster 2 (Figure 2a, Table 1). Clusters 2 and 3 were also not significantly different from one another for synonymous π (paired t tests, t_6_ = -1.39, p = 0.213). Both clusters 1 and 2 were significantly different from cluster 3 for both synonymous and non-synonymous estimates of θ_W_ (paired t tests, synonymous θ_W_: 1 vs 3: t_6_ = 5.219, p = 0.002; 2 vs 3: t_6_ = 2.549, p = 0.044; non-synonymous θ_W_: 1 vs 3: t_6_ = 3.762, p = 0.009; 2 vs 3: t_6_ = 2.545, p = 0.044). However, they were not significantly different from each another (paired t tests, synonymous θ_W_: 1 vs 2: t_6_ = -1.366, p = 0.221; non-synonymous θ_W_: 1 vs 2: t_6_ = 0.698, p = 0.511).

**Table 1:** **The mean number of segregating sites and estimates of synonymous and non-synonymous genetic diversity for each cluster across the seven genetic regions**

	**Synonymous sites**				**Non-synonymous sites**			
**Cluster**	**Sites** ^**1**^	**SS** ^**2**^	**π** ^**3**^	**θ**_**w**_^**4**^	**Sites** ^**1**^	**SS** ^**2**^	**π** ^**3**^	**θ**_**w**_^**4**^
1	95.6 (3.0)	11.1 (4.3)	0.0131 (0.0047)	0.0196 (0.0079)	315.4 (19.8)	5.4 (1.8)	0.0008 (0.0005)	0.0028 (0.0008)
2	95.6 (3.1)	11.4 (10.8)	0.0379 (0.0477)	0.0354 (0.0346)	315.4 (19.7)	2.7 (1.6)	0.0015 (0.0011)	0.0025 (0.0013)
3	96.3 (3.0)	1.6 (1.1)	0.0056 (0.0044)	0.0046 (0.0034)	314.7 (19.1)	0.9 (1.2)	0.0008 (0.0010)	0.0008 (0.0011)

^1^The average number of sites per genetic region that were classified as either synonymous or non-synonymous respectively.

^2^The average number of segregating sites across the seven genetic regions.

^3^The average pairwise difference per site [18,19].

^4^Watterson’s estimator of genetic diversity based on the number of segregating sites [20]**.**

() Standard deviations are indicated in parentheses.

To estimate effective population size we utilized the formula: N_e_ = θ/2 μ, where N_e_ is the effective population size, θ is a measure of per locus diversity and μ is the per locus mutation rate [20,21]. A mutation rate is not currently known for *A. phagocytophilum*. Therefore, to estimate effective population sizes we utilized an average of previously reported mutation rates for other bacterial species (~0.003 per genome [22]). The *A. phagocytophilum* genome is approximately 1.4 Mb in length [23]. Therefore we estimated the per locus mutation rate as ~2 × 10^−9^, with the assumption that it is the same for all three strains. We calculated effective population size using both synonymous measures of genetic diversity (θ_W_ and π). The effective population size of the cluster 1 strain was estimated to be between 3.28 × 10^6^ and 4.90 × 10^6^, the cluster 2 strain to be between 8.85 × 10^6^ and 9.48 × 10^6^, and the cluster 3 strain to be between 1.15 × 10^6^ and 1.40 × 10^6^.

It is possible that variation in our sampling efforts could affect estimates of genetic diversity. However, we saw the overall highest mean diversity levels in the cluster 2 strain, which had the smallest sample size (n = 18). Nonetheless, we wanted to determine if the much larger sample size of cluster 1 could have influenced our estimates of genetic diversity in this strain. To test this we randomly drew 20 samples from the full data set with replacement and calculated the four diversity statistics for this subset of the data. We did this 200 times to generate bootstrapped confidence intervals of our estimates. Based on this analysis, the mean synonymous π for cluster 1 was 0.0130 (±0.0075), mean synonymous θ_W_ was 0.0163 (±0.0067), mean non-synonymous π was 0.0008 (±0.0005), and mean non-synonymous θ_W_ was 0.0013 (±0.0008). None of these results differed significantly from the same diversity estimate based on the full dataset (data not shown).

### Linkage disequilibrium

**Figure 3:**
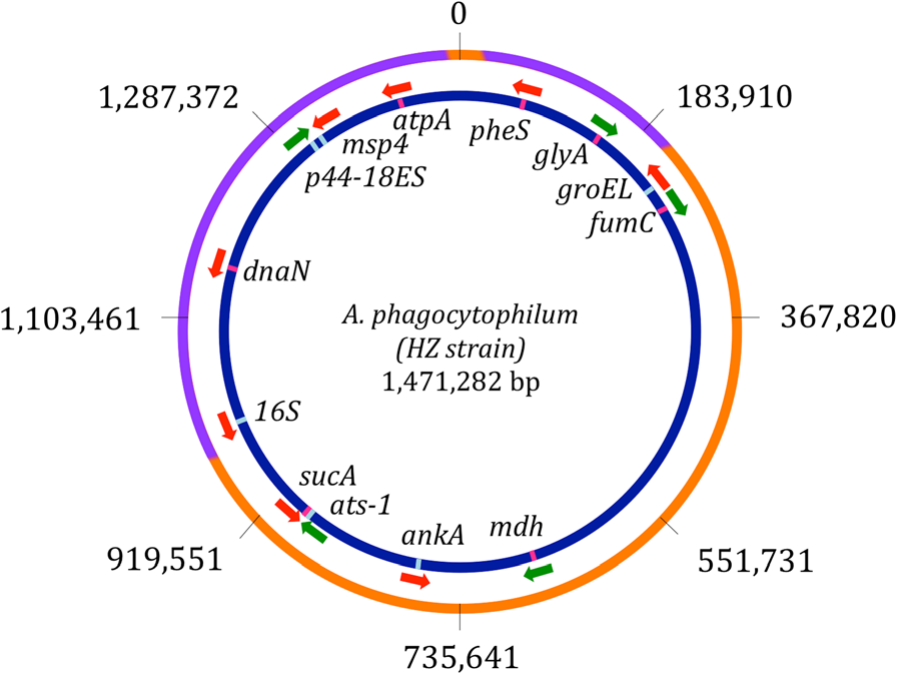
**A representation of the circular genome of** ***A. phagocytophilum*** **based on the sequenced HZ strain (NC_007797.1, [** 23 **]).** The outer circle (orange & purple) gives genome landmarks in base pairs. The two purple sections indicate the locations of two *p44* pseudogene clusters [65]. The inner circle (dark blue) shows the location of the seven genetic regions used in this study (pink bars) as well as six other genetic regions that have been important for *A. phagocytophilum* strain characterization or may have functional importance in host interactions (light blue bars). Green arrows indicate that the coding sequence of a genetic region is in the forward direction relative to the published genome and red arrows indicate that the coding sequence of the genetic region is in the reverse direction relative to the published genome. (See Additional file 1: Table S5 for more details).

The differences in the amount of standing genetic diversity observed within the three *A. phagocytophilum* strains suggest that selection and/or demography has differed between them. In recombining organisms the effects of selection are expected to occur locally, whereas the effects of demographic change should be observable genome wide. However, in clonal organisms such as bacteria, selection will affect diversity across the entire genome unless recombination breaks up linkages between genomic regions [24]. While such recombination was once considered rare in bacteria, increasing evidence suggests that it can occur at a sufficient rate to minimize linkages among loci [25,26]. The seven genetic regions in this study are distributed broadly across the *A. phagocytophilum* genome, making it possible that they have evolved at least semi-independently (Figure 3). Furthermore, homologous recombination plays a large roll in allowing *A. phagocytophilum* populations to avoid immune defenses and adapt to specific hosts [27]. This may also reduce the influence of linkage on genetic diversity patterns in this bacterium.

We quantified the extent of inter-locus linkage disequilibrium (LD) to assess how influential linkage may be on patterns of diversity among these strains. To do this we calculated a variant of the index of association called r_D_ [24,28], using all genetic regions for each cluster. This statistic measures whether two individuals being similar at one locus makes them more likely to be similar at another locus. It ranges from 0 to 1, and a value significantly different from 0 indicates that recombination has been rare and loci may be in LD. For the full datasets, r_D_ was significantly different from 0 in clusters 1 and 3 (Cluster 1: n = 227, r_D_ = 0.126, p < 0.001; Cluster 3: n = 20, r_D_ = 0.256, p = 0.001). Cluster 2 did not have a r_D_ value statistically different from 0 (n = 18, r_D_ = 0.078, p = 0.528). These results suggest that the genetic regions used in our analyses from clusters 1 and 3 are not independent and that the different influences of selection and demography may be obscured. However, as Maynard Smith and colleagues pointed out, within a bacterial population it is common for one or a few genotypes to occasionally arise in a population and rapidly become widespread [24]. Depending on the speed at which this occurs, there may not be sufficient time for recombination to break up linkage groups and population samples may be comprised of representatives from a small number of clones. This may be especially true when sampling efforts are uneven for various geographic regions or hosts. To negate some of this problem, it was suggested that identical samples be collapsed into a single representative sample [24]. When we reduced the dataset for clusters 1 and 3 to only unique samples, we found that r_D_ was no longer significantly different from 0 for cluster 3 (n = 10, r_D_ = 0.176, p = 0.170). However, cluster 1 still showed evidence of LD (n = 10, r_D_ = 0.021, p < 0.001).

### Selection

**Figure 4:**
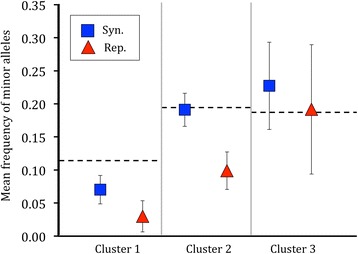
**The mean population frequency of minor alleles for synonymous and non-synonymous segregating sites.** The horizontal dashed lines represent the expected mean under neutrality given the number of observed segregating sites [34]. Error bars indicate 95% confidence intervals based on 10,000 bootstrap replicates of the observed data, randomly sampled with replacement.

To look at the varying influences of selection and demography between these three strains, we first examined the average frequency of minor alleles segregating in each population (Figure 4). In all three strains alleles at non-synonymous sites were on average segregating at a lower frequency than synonymous minor alleles. In cluster 1, both non-synonymous and synonymous mutations were segregating at lower frequencies than would be predicted under strict neutrality. In contrast, the cluster 3 strain exhibited a higher average allele frequency for synonymous sites than the neutral expectation, although the confidence intervals overlap this expectation. In cluster 2 only the frequency of non-synonymous mutations differed from the neutral expectation.

We next compared d_N_/d_S_ ratios for each locus [29]. Average d_N_/d_S_ was highest for cluster 2 and lowest for cluster 3 (Table 2). Clusters 2 and 3 were significantly different from one another (paired t test, t_6_ = 2.476, p = 0.048), but no other pairwise comparison between clusters was statistically different (paired t tests, 1 vs. 2: t_6_ = -1.138, p = 0.299; 1 vs 3: t_6_ = 1.341, p = 0.228). None of the seven genetic regions in any of the three strains had a d_N_/d_S_ > 1 (Additional file 1: Table S2). These results are consistent with the lower average allele frequency observed for non-synonymous sites compared to synonymous sites.

**Table 2:** **Average estimates for tests of selection and additional neutrality tests based on the observed number of segregating sites**

**Cluster**	**d**_**N**_ **/d**_**S**_^**1**^	**DoS** ^**2**^	**Tajima’s D**	**Fu & Li’s D**	**Fay & Wu’s H**
1	0.078 (0.054)	−0.149 (0.181)	−1.129 (0.508)	−0.972 (0.596)	−4.400* (2.153)
2	0.086 (0.047)	−0.009 (0.112)	−0.585 (0.990)	−0.337 (1.169)	−1.931 (4.100)
3	0.070 (0.048)	−0.026 (0.325)	0.337 (1.013)	0.677 (0.328)	−1.079* (1.539)

^1^The average ratio of non-synonymous substitutions per non-synonymous site (d_N_) to synonymous substitutions per synonymous site (d_S_) for the seven loci.

^2^The average estimate from the direction of selection (DoS) test.

*Indicates statistical significance based on 10,000 coalescent simulations with the number of observed segregating sites and no recombination (p < 0.05).

() Standard deviations are indicated in parentheses.

We used the McDonald-Kreitman test (MK test) to further look for signatures of selection in each locus within the three clusters [30]. Specifically, we compared the observed levels of polymorphism and divergence at synonymous and non-synonymous sites to look for deviations from neutral expectations in any loci. Such deviations could be the result of adaptive processes, or they may indicate the presence of slightly deleterious mutations segregating in the population [31]. Only three genes among any of the strains exhibited significant deviations from neutrality (Additional file 1: Table S2; Cluster 1 (*atpA*): Fisher’s exact test, p < 0.001; Cluster 2 (*pheS*): Fisher’s exact test p = 0.026; Cluster 3 (*fumC*): Fisher’s exact test, p = 0.008.). To determine the direction of these deviations we used a variant of the neutrality index called the direction of selection test (DoS), which corrects for potential biases when the amount of data is small [32,33]. A positive DoS suggests that positive selection has acted on a region, whereas a negative DoS indicates that slightly deleterious alleles may be segregating. One gene from cluster 2 had both a positive DoS value and significantly deviated from neutrality based on the MK test (*phes,* DoS = 0.23). This indicates that positive selection has likely acted to produce divergence in this gene. The other loci that had significant deviations from neutrality had negative DoS values (Cluster 1 (*atpA*) DoS = -0.54; Cluster 3 (*fumC*) DoS = -0.68). Negative DoS values indicate an excess of non-synonymous polymorphism, which can occur when slightly deleterious mutations are circulating in a population.

### Demography

While we found evidence for both positive selection and segregating deleterious alleles, purifying selection appears to be the primary selective force acting in all three strains. However, differences in average segregating site frequency between strains suggest that selection has not been the only factor influencing genetic diversity levels. To investigate the potential effects of demographic changes within these populations, we examined three complimentary population statistics that compare observed segregating site frequencies to expectations under neutrality. These were: Tajima’s D [34], Fu & Li’s D [35] and Fay & Wu’s H [36]. Combining multiple statistics in this fashion gives a more clear picture of the processes acting on a population than any one test alone [37,38].

Tajima’s D compares the number of segregating sites in the population sample to genetic diversity (π). Under neutrality these two numbers should be very similar and D will be approximately 0. When D is greater than 0 it indicates that there is a high level of intermediate frequency polymorphism relative to neutral expectations. Conversely, if D is less than 0 it indicates an excess of low frequency polymorphism relative to neutral expectations. The average value of D was negative for both clusters 1 and 2, but positive for cluster 3 (Table 2). However, these deviations from 0 were not significant for any strain (data not shown). For both clusters 1 and 2, two of the seven loci were significantly negative, suggesting that purifying selection may have acted on these genetic regions (Additional file 1: Table S3).

Fu & Li’s D is similar to Tajima’s D except that it specifically compares the number of mutations observed in just one population member (‘singletons’) to the expected number under neutrality. This makes the test more sensitive to selective sweeps, which are predicted to be a powerful force in bacterial evolution [24]. Fu & Li’s D can also be useful for detecting bottlenecks [37]. The average value of Fu & Li’s D was not significant for any of the three strains (data not shown). Fewer individual loci had significant deviations from the neutral expectation than were observed for Tajima’s D as well, and only one locus in cluster 1 was significant for Fu & Li’s D, but not Tajima’s D (Additional file 1: Table S3).

The third test, Fay & Wu’s H, compares the number of high-frequency derived mutations to those at intermediate frequency. This test was designed specifically to detect a selective sweep, as linked sites should rise in frequency around the target of positive selection, increasing derived allele frequencies. H is less sensitive to population expansion than the other two tests [36]. Both clusters 1 and 3 had an average negative H value significantly different from 0, indicating an excess of high frequency, derived alleles. For cluster 1, four of the seven loci had a significantly negative H value (Additional file 1: Table S3). Two loci out of seven were significantly negative for cluster 2 and two out of six were significant for cluster 3. Overall, a large proportion of H values were negative throughout the three strains, indicating a prevalence of high frequency, derived segregating alleles.

## Discussion

*A. phagocytophilum* in Europe circulates in multiple, discreet enzootic cycles and consequently distinct populations of the bacterium have been identified [13-15]. One strain infects a wide array of mammalian hosts including humans, livestock and other domestic animals. In contrast, a second strain specializes on roe deer. A third strain infects rodents and insectivores, and differs in the tick vector that facilitates transmission between hosts [13]. Among host-dependent bacteria such as *A. phagocytophilum*, transmission opportunities between competent hosts may occur less frequently for host specialists [2]. This may act to limit their potential for population growth. A lower density of prospective hosts in the community may also limit effective population sizes. Conversely, specialization may facilitate adaptation to competent hosts and allow greater within*-*host population densities [1]. This may support larger effective population sizes in host specialists. In accordance with this second hypothesis, the roe deer specialist (cluster 2) had the largest estimated effective population size of the three strains. Specific adaptations for colonizing roe deer may allow this strain to reach higher within-host population densities compared to generalist strains [1]. Additionally, roe deer represent a very large host pool that likely increases *A. phagocytophilum* rates of encounter and allows high densities of this specialized strain to be maintained within ecological communities [4,39]. Higher *A. phagocytophilum* prevalence rates in roe deer compared to other hosts suggests that infection may be chronic in these animals or that frequent reinfection may occur. In either case, higher effective population sizes would be achieved.

The cluster 1 strain, which is a host generalist, had a smaller estimated effective population size than the roe deer specialist. It is likely that this strain of *A. phagocytophilum* is not as well adapted to any particular host. Therefore, it achieves lower within-host population densities than the specialist strain. Lower densities could reduce the rate of transmission between hosts. While increasing the number of species a pathogen can utilize produces more infected individuals throughout an ecological community, for any particular species the proportion of individuals infected will be smaller [2]. This results in less frequent transmission events and fewer opportunities for adaptive evolution to occur within a population.

The cluster 3 strain had a much smaller effective population size than either the cluster 1 or 2 strains. This strain’s primary hosts are insectivores and rodents, which have the highest population densities of any mammal in Europe [40]. However, the cluster 3 strain is predominately transmitted by the nest-living tick, *I. trianguliceps*, resulting in a distinct zoonotic cycle with minimal overlap in either host or vector with the other *A. phagocytophilum* strains [13,41]. Furthermore, evidence suggests that both voles and shrews may be able to clear *A. phagocytophilum* infection as indicated by its absence in winter months when tick vectors are dormant [13,42]. By contrast, roe deer are found to harbor infections year round [13]. It is therefore probable that vector population dynamics play a larger role in limiting the effective population size of this strain than in other strains [42]. Challenges related to host adaptation may also be a factor as insectivores and rodents are highly diverged evolutionarily [16].

Both demographic events and selection have acted to produce the effective population sizes of these strains. In all three strains, minor alleles at non-synonymous sites were on average segregating at a lower frequency than synonymous minor alleles. This observation suggests that purifying selection has been a strong force acting on non-synonymous variation in these populations [34,43-45]. A predominance of purifying selection in housekeeping genes is typical for pathogenic bacterial species [46,47]. However, we also found evidence for differences in demographic and selection history between the three populations in this study.

The cluster 1 strain harbored a large number of low-frequency variants in each of the genetic regions analyzed. An overall excess of low frequency alleles may be an indication that this population has expanded in size [34,48-50]. It could also indicate that selective sweeps have occurred in this strain, followed by new mutations entering the population. We additionally found an excess of high-frequency derived alleles as indicated by negative Fay and Wu’s H values for all genes. A negative H typically occurs only in incidences of a selective sweep and is a primary way to distinguish sweeps from population expansion [36]. Finally, the cluster 1 strain had a significantly high level of LD between loci, even when identical clones were excluded from the analysis. Selective sweeps are predicted to increase LD, whereas population expansion decreases LD [49,51]. Based on these results, we conclude that selective sweeps in this strain have been important contributors to genetic divergence between this and other *A. phagocytophilum* populations. These sweeps would have allowed neutral and mildly deleterious alleles to rise in frequency and fix in the genome through genetic hitchhiking.

Only the frequency of non-synonymous mutations differed from the neutral expectation in the cluster 2 strain. We also found that cluster 2 had the most variable diversity levels between genetic regions. Finally, there was no evidence of LD between loci in this strain. Together, these results indicate that neither demographic changes, nor genome-wide selection events, have likely affected patterns of diversity in this strain. Rather, it appears that selection acting locally in the genome has had the greatest influence on strain-level genetic diversity. Several of the loci in this study appear to have been influenced by local selective sweeps as evidenced by negative values for all three demographic statistics. Another locus had the opposite trend, with positive values for all three neutrality tests, indicating that most segregating sites were at intermediate frequencies. This can occur if balancing selection is acting on a region or else there is unrecognized population structure. Interestingly, this was the only locus that was both significant for the MK test and that had a positive DoS. These observations strengthen the hypothesis that balancing selection has acted on this locus or in a region closely linked to it. Ultimately, it appears that a relatively large effective population size and frequent recombination has allowed selection to operate locally in the genome of this strain without affecting genetic diversity more broadly. It also means that stochastic changes in population size are unlikely to have had a major influence on the establishment of divergence between this and other strains. Rather mutation and selection are likely to be the primary drivers of divergence in this *A. phagocytophilum* population.

The cluster 3 strain had a higher than expected average allele frequency for synonymous segregating sites, suggesting that there is a deficiency of low-frequency segregating alleles in this population. Such a deficiency can arise due to genetic bottlenecks when low-frequency alleles are disproportionally lost as the size of the population decreases [34]. A high proportion of positive Tajima’s D and Fu & Li’s D values among cluster 3 loci also support the conclusion that this population has fewer low frequency segregating alleles than expected, and that it likely experienced one or more bottlenecks. Finally, we see extensive variance in diversity levels between loci in this strain. Increased variance between genetic regions is expected after a population reduction [52]. Hidden population structure and balancing selection can also cause genetic patterns similar to a bottleneck. However, both of these factors should increase overall genetic diversity, whereas the cluster 3 strain was found to have the lowest amount of genetic diversity among the three strains. If one or more bottlenecks have occurred in this population, it is likely that many segregating alleles were fixed by genetic drift. This may have produced extensive divergence between this and other *A. phagocytophilum* strains. Of the three strains examined in this study, the cluster 3 strain is the most divergent (Figure 1, Table 2). Recent bottlenecks may also have contributed to the smaller effective population size we observed in this strain.

Interestingly, despite the large differences in the effective population sizes of these three strains, their d_N_/d_S_ ratios were relatively more similar (Table 3). While synonymous diversity levels are indicative of recent selection and demographic events, d_N_/d_S_ ratios are more reflective of long-term evolutionary history [53]. The similarity of d_N_/d_S_ ratios observed between the three strains may indicate that these populations historically had more similar effective population sizes. Furthermore, while theory predicts that populations with smaller effective population sizes should have larger d_N_/d_S_ ratios [54], in this study we see the opposite trend (Table 2). The reasons for this observation are unclear, but the theoretical prediction is based on an assumption that most non-synonymous mutations are deleterious. If a population is adapting to a new host or other conditions, non-synonymous mutations may be selected for, increasing the d_N_/d_S_ ratio.

**Table 3:** **Pairwise divergence estimates for the three clusters based on the concatenated dataset of all seven genetic regions**

**Clusters**	**Fst** ^**1**^	**D**_**xy**_^**2**^	**D**_**a**_^**3**^
1 vs 2	0.757	0.027	0.021
1 vs 3	0.972	0.100	0.097
2 vs 3	0.945	0.105	0.099

^1^Fixation index [59,60].

^2^The average number of nucleotide substitutions per site between each cluster [19].

^3^The net number of nucleotide differences per site between each cluster [19].

In addition to host range, other factors have undoubtedly influenced genetic diversity and divergence in these populations as well. For example, in *A. phagocytophilum* regular recombination of *p44* surface genes and functional pseudogenes allows populations within a vertebrate host to evade immune responses [27,55]. The *p44* expression cassettes and pseudogenes can be found throughout the genome, although there are two regions where the majority of these sites cluster (Figure 3). Therefore, selection from host immune defenses could influence both host adaptation and recombination frequency, which could potentially affect patterns of genetic diversity throughout the genomes of these populations. Other host-specific characteristics could also play a role in limiting the effective population sizes of these strains, as could the population dynamics of transmission vectors. Vector biology may be particularly important in limiting the effective population size of the cluster 3 strain. Unrecognized population structure in these strains may also have contributed to observed patterns of genetic diversity [56]. Additional work will be required to determine what factors have been the most important in influencing genetic diversity and divergence in *A. phagocytophilum*.

## Conclusions

Our analyses reveal that evolutionary processes acting on host-adapted *A. phagocytophilum* strains have been influenced by their effective population size, which in turn has likely been impacted by the ecology and population densities of competent hosts. It remains to be determined what factors contributed to the initial production of host range differences between these strains, but both vector and host population dynamics have likely played important roles [57]. Specialization alone has not restricted population growth in *A. phagocytophilum*, but rather may have facilitated relative increases in effective population size. Frequent homologous recombination in some strains, possibly in conjunction with evolving responses to immune defense, has likely reduced the impact of genetic linkage between genome regions and has allowed adaptive processes to occur in these bacteria without impacting genome-wide genetic diversity. However, in other cases bottlenecks have likely reduced genetic diversity and may have restricted adaptation rates. Such population reductions may also have allowed for drift to contribute to divergence between strains.

Pathogens with a broad host range have the greatest probability of being transmitted to humans [58]. This appears to have been the case for *A. phagocytophilum* where it is the generalist strain that is found to infect people in Europe [14]. Overall, better knowledge of how the life history characteristics of natural hosts influence bacterial population dynamics will provide insights into the maintenance of genetic diversity in emerging zoonotic bacteria. Understanding this diversity will be important for predicting the potential of such bacteria to emerge as prospective zoonotic agents as they evolve in response to ever changing host population dynamics.

## Methods

### Data set

For this study, we utilized partial sequences of seven *A. phagocytophilum* genetic regions totaling 2,877 base pairs. These sequences were isolated from 17 different host mammals and *I. ricinus* ticks (Additional file 1: Tables S4, S5). Using maximum-likelihood phylogenetic analysis, Huhn and colleagues showed that these samples could be clustered into one of three genetically distinct groups [14]. These likely represent unique populations with independent transmission cycles. We followed these same cluster classifications for the samples in this analysis. From the original dataset, we removed all but one set of sequences in cases where there were multiple temporal samples from a single host. We also removed sequences that were isolated in the United States and all sequences from any sample harboring polymorphic regions in any of the seven loci, as this indicates the host may have been infected with multiple clones. This reduced dataset left us with 227 samples in cluster 1, 18 samples in cluster 2, and 20 samples in cluster 3. To further assess the extent of divergence among these three populations, we calculated three pairwise measures of genetic divergence between the clusters: the fixation index (Fst, [59,60]), the average number of nucleotide substitutions per site between each cluster (D_xy_, [19]) and the net number of nucleotide differences per site between each cluster (D_a_, [19]). These were calculated using concatenated datasets across all sites with the program DnaSP [61]. Our results confirmed previous findings that these three strains are highly diverged from one another (Table 3).

### Genetic diversity

For each strain, we calculated two measures of genetic diversity, the average pairwise nucleotide diversity per site (π, [18,19]) and Watterson’s θ (θ_w_), which is based on the number of segregating sites [20]. For both measurements, synonymous and non-synonymous diversity was calculated separately using the program Polymorphorama [45]. For π, calculations were based on the number of mutations when more than two alleles were segregating at a site. To assess if any cluster was statistically different from another for any diversity measure and site class, we used a paired t-test as implemented in the program R [62].

Additionally, because of its much larger sample size, we examined whether the cluster 1 samples would exhibit similar levels of genetic diversity to the full data set when a smaller set of samples was examined. To do this, we randomly selected 20 of the samples from the full dataset (with replacement) and again calculated the same diversity statistics using Polymorphorama [45]. We repeated this 200 times to determine confidence intervals.

### Linkage disequilibrium

To examine inter-locus recombination, for each strain we calculated r_D_ as implemented in the program MultiLocus (ver. 1.2.2, [28]). Statistical significance was determined by comparing 1,000 randomized datasets with a null hypothesis of complete linkage equilibrium between loci (r_D_ = 0). r_D_ was calculated for all three clusters using all samples. r_D_ was also calculated for clusters 1 and 3 using reduced datasets where all but one representative of identical clones was removed.

### Selection

For each population we determined the average frequency of segregating alleles for both synonymous and non-synonymous sites. Allele frequencies were determined using the program Polymorphorama [45]. The expected neutral mean frequency for segregating alleles was calculated based on the sample size and number of observed segregating sites [34].

For all tests of selection, non-synonymous sites (selected class) were compared to synonymous sites (neutral class). We first compared the number of non-synonymous changes per non-synonymous site to synonymous changes per synonymous site (d_N_/d_S_) for each locus [29]. Ratios greater than one suggest that positive selection has acted to generate divergence between populations. Ratios less than one suggest that purifying selection has been the more common selective force, eliminating disadvantageous amino acid substitutions as they arose, but allowing for synonymous changes between populations to fix. To count the number of synonymous and non-synonymous sites as well as divergences, we used the program Polymorphorma [45]. We also performed the McDonald-Kreitman test in each cluster for each locus to examine evidence of positive selection [30]. For each locus in each strain the population data was compared to an outgroup sequence. For clusters 1 and 2 we used a consensus sequence from the cluster 3 data, and for cluster 3 we used a consensus sequence from the cluster 1 data. Statistical significance was determined using a two-tailed Fisher’s exact test [63] as implemented in R [62]. A variant of the neutrality index, the direction of selection test (DoS) was used to determine the direction of deviation from neutrality in each loci [32,33].

### Demography

We used DnaSP to calculate each of our demographic statistics using all sites in each loci [61]. These statistics were: Tajima’s D [34], Fu & Li’s D [35] and Fay & Wu’s H [36]. Fu & Li’s D and Fay & Wu’s H require the use of an outgroup to distinguish ancestral and derived alleles. For clusters 1 and 2, we used a consensus sequence from the cluster 3 data to polarize segregating sites. For cluster 3 we used a consensus sequence from the cluster 1 data. Statistical significance was determined for all demographic estimates by simulating 10,000 replicates of the standard neutral model based on the number of segregating sites with no recombination. For Tajima’s D and Fay and Wu’s H these simulations were carried out in the program *ms* [64]. For Fu & Li’s D, simulations were carried out in DnaSP [61].

## Supporting data

The data set supporting the results of this article are available from the *Anaplasma phagocytophilum* MLST database, [http://pubmlst.org/aphagocytophilum/], and on Genbank (GenBank accession numbers KF242733 through KF245413, see Additional file 1: Table S4 for more information on individual samples).

## Additional file

Additional file 1: Table S1. The number of segregating sites and mutations as well as estimates of synonymous and non-synonymous genetic diversity for each genetic region individually and for all the sequences as a whole. **Table S2.** Counts for the number of divergent and segregating sites for each genetic region in each strain, plus estimates of selection parameters for individual locus and all loci together. **Table S3.** Calculations of neutrality statistics for each genetic region from each cluster. **Table S4.** Samples used in this study (GenBank accession numbers KF242733 through KF245413, [14]). **Table S5.** The location of the seven genetic regions used in this study in the *A. phagocytophilum* genome, in addition to six other regions that have been important for characterizing populations or that play a role in host interactions (*groEL*, *ankA*, *ats*-1*, 16S, msp4, p44-18ES*). Additionally, the locations of the two primary clusters of *p44* pseudogenes are given.

## References

[1] Toft C, Andersson SGE (2010). Evolutionary microbial genomics: insights into bacterial host adaptation. Nat Rev.

[2] Roche B, Dobson AP, Guégan J-F, Rohani P (2012). Linking community and disease ecology: the impact of biodiversity on pathogen transmission. Phil Trans R Soc B.

[3] Leggett HC, Buckling A, Long GH, Boots M (2013). Generalism and the evolution of parasite virulence. Trends Ecol Evol.

[4] Stuen S, Granquist EG, Silaghi C (2013). *Anaplasma phagocytophilum*- a widespread multi-host pathogen with highly adaptive strategies. Front Cell Infect Microbiol.

[5] Lajeunesse MJ, Forbes MR (2002). Host range and local parasite adaptation. Proc R Soc Lond B.

[6] Pugliese A (2011). The role of host population heterogeneity in the evolution of virulence. J Biol Dyn.

[7] Woolfit M, Bromham L (2003). Increased rates of sequence evolution in endosymbiotic bacteria and fungi with small effective population sizes. Mol Biol Evol.

[8] Leffler EM, Bullaughey K, Matute DR, Meyer WK, Ségurel L, Venkat A (2012). Revisiting an old riddle: what determines genetic diversity levels within species?. PLoS Biol.

[9] Rego ROM, Bestor A, Štefka J, Rosa PA (2014). Population bottlenecks during the infectious cycle of the Lyme disease spirochete *Borrelia burgdorferi*. PLoS One.

[10] Shapiro BJ, Friedman J, Cordero OX, Preheim SP, Timberlake SC, Szabó G (2012). Population genomics of early events in the ecological differentiation of bacteria. Science.

[11] Lenski RE, Travisano M (1994). Dynamics of adaptation and diversification: A 10,000-generation experiment with bacterial populations. Proc Natl Acad Sci U S A.

[12] Lang GI, Rice DP, Hickman MJ, Sodergren E, Weinstock GM, Botstein D (2013). Pervasive genetic hitchhiking and clonal interference in forty evolving yeast populations. Nature.

[13] Bown KJ, Lambin X, Ogden NH, Begon M, Telford G, Woldehiwet Z (2009). Delineating *Anaplasma phagocytophilum* ecotypes in coexisting, discrete enzootic cycles. Emerg Infect Dis.

[14] Huhn C, Winter C, Wolfsperger T, Wüppenhorst N, Strašek Smrdel K, Skuballa J (2014). Analysis of the population structure of *Anaplasma phagocytophilum* using multilocus sequence typing. PLoS One.

[15] Van Der Giessen J, Takken W, Van Wieren SE, Takumi K, Sprong H (2014). Circulation of four *Anaplasma phagocytophilum* ecotypes in Europe. Parasit Vectors.

[16] Meredith RW, Janečka JE, Gatesy J, Ryder OA, Fisher CA, Teeling EC (2011). Impacts of the cretaceous terrestrial revolution and KPg extinction on mammal diversity. Science.

[17] Ishiguro H, Ichihar Y, Namikawa T, Nagatsu T, Kurosawa Y (1989). Nucleotide sequence of *Suncus murinus* immunoglobulin *μ* gene and comparison with mouse and human *μ* genes. FEBS Lett.

[18] Nei M, Li WH (1979). Mathematical model for studying genetic variation in terms of restriction endonucleases. Proc Natl Acad Sci.

[19] Nei M (1987). Molecular evolutionary genetics.

[20] Watterson GA (1975). On the number of segregating sites in genetical models without recombination. Theor Popul Biol.

[21] Kimura M, Crow JF (1964). The number of alleles that can be maintained in a finite population. Genetics.

[22] Drake JW, Charlesworth B, Charlesworth D, Crow JF (1998). Rates of spontaneous mutation. Genetics.

[23] Dunning Hotopp JC, Lin M, Madupu R, Crabtree J, Angiuoli SV, Eisen J (2006). Comparative genomics of emerging human ehrlichiosis agents. PLoS Genet.

[24] Maynard Smith J, Smith NH, O’Rourke M, Spratt BG (1993). How clonal are bacteria?. Proc Natl Acad Sci U S A.

[25] Feil EJ, Holmes EC, Bessen DE, Chan M-S, Day NPJ, Enright MC (2001). Recombination within natural populations of pathogenic bacteria: short-term empirical estimates and long-term phylogenetic consequences. Proc Natl Acad Sci.

[26] Vos M, Didelot X (2009). A comparison of homologous recombination rates in bacteria and archaea. ISME J.

[27] Rikihisa Y (2010). *Anaplasma phagocytophilum* and *Ehrlichia chaffeensis*: subversive manipulators of host cells. Nat Rev Microbiol.

[28] Agapow P-M, Burt A (2001). Indices of multilocus linkage disequilibrium. Mol Ecol Notes.

[29] Miyata T, Yasunaga T (1980). Molecular evolution of mRNA: a method for estimating evolutionary rates of synonymous and amino acid substitutions from homologous nucleotide sequences and its applications. J Mol Evol.

[30] McDonald JH, Kreitman M (1991). Adaptive protein evolution at the *Adh* locus in *Drosophila*. Nature.

[31] Ohta T (1993). Amino acid substitution at the Adh locus of *Drosophila* is facilitated by small population size. Proc Natl Acad Sci U S A.

[32] Rand DM, Kann A (1996). Polymorphims in mitochondrial DNA: contrasts among genes from *Drosophila*, mice, and humans. Mol Biol Evol.

[33] Stoletzki N, Eyre-Walker A (2011). Estimation of the neutrality index. Mol Biol Evol.

[34] Tajima F (1989). Statistical method for testing the neutral mutation hypothesis of DNA polymorphism. Genetics.

[35] Fu Y-X, Li WH (1993). Statistical tests of neutrality of mutations. Genetics.

[36] Fay JC, Wu CI (2000). Hitchhiking under positive Darwinian selection. Genetics.

[37] Depaulis F, Mousset S, Veuille M (2003). Power of neutrality tests to detect bottlenecks and hitchhiking. J Mol Evol.

[38] Ramírez-Soriano A, Ramos-Onsins SE, Rozas J, Calafell F, Navarro A (2008). Statistical power analysis of neutrality tests under demographic expansions, contractions and bottlenecks with recombination. Genetics.

[39] Burbaitė L, Csányi S (2009). Roe deer population and harvest changes in Europe. Est J Ecol.

[40] Krebs CJ (2013). Population fluctuations in rodents.

[41] Blaňarová L, Stanko M, Carpi G, Miklisová D, Víchová B, Mošanský L (2014). Distinct *Anaplasma phagocytophilum* genotypes associated with *Ixodes trianguliceps* ticks and rodents in Central Europe. Tick Tick-Borne Dis.

[42] Bown KJ, Begon M, Bennett M, Woldehiwet Z, Ogden NH (2003). Seasonal dynamics of *Anaplasma phagocytophilum* in a rodent-tick (*Ixodes trianguliceps*) system, United Kingdom. Emerg Infect Dis.

[43] Akashi H (1999). Within- and between-species DNA sequence variation and the ‘footprint’ of natural selection. Gene.

[44] Andolfatto P (2005). Adaptive evolution of non-coding DNA in Drosophila. Nature.

[45] Haddrill PR, Bachtrog D, Andolfatto P (2008). Positive and negative selection on noncoding DNA in *Drosophila simulans*. Mol Biol Evol.

[46] Dingle KE, Colles FM, Wareing DRA, Ure R, Fox AJ, Bolton FE (2001). Multilocus sequence typing system for *Campylobacter jejuni*. J Clin Microbiol.

[47] Feil EJ, Cooper JE, Grundmann H, Robinson DA, Enright MC, Berendt T (2003). How clonal is *Staphylococcus aureus*?. J Bacteriol.

[48] Slatkin M, Hudson RR (1991). Pairwise comparisons of mitochondrial DNA sequences in stable and exponentially growing populations. Genetics.

[49] Braverman JM, Hudaon RR, Kaplan NL, Langley CH, Stephan W (1995). The hitchhiking effect on the site frequency spectrum of DNA polymorphism. Genetics.

[50] Fu Y-X (1997). Statistical tests of neutrality against population growth, hitchhiking and background selection. Genetics.

[51] Przeworski M (2002). The signature of positive selection at randomly chosen loci. Genetics.

[52] Carson HL (1990). Increased genetic variance after a population bottleneck. Trends Ecol Evol.

[53] Batut B, Knibbe C, Marais G, Daubin V (2014). Reductive genome evolution at both ends of the bacterial population size spectrum. Nat Rev Microbiol.

[54] Ohta T (1992). The nearly neutral theory of molecular evolution. Annu Rev Ecol Syst.

[55] Rejmanek D, Foley P, Barbet A, Foley J (2012). Evolution of antigen variation in the tick-borne pathogen *Anaplasma phagocytophilum*. Mol Biol Evol.

[56] Feil EJ, Robinson DA, Falush D, Feil EJ (2010). Linkage, selection and the clonal complex. Bacterial population genetics in infectious disease.

[57] Schmid-Hempel P (2011). Evolutionary Parasitology.

[58] Taylor LH, Latham SM, Woolhouse MEJ (2001). Risk factors for human disease emergence. Phil Trans R Soc B.

[59] Wright S (1951). The genetical structure of populations. Ann Eugenics.

[60] Lynch M, Crease TJ (1990). The analysis of population survey data on DNA sequence variation. Mol Biol Evol.

[61] Librado P, Rozas J (2009). DnaSP v5: A software for comprehensive analysis of DNA polymorphism data. Bioinformatics.

[62] R Core Team (2014). R: a language and environment for statistical computing.

[63] Fisher RA (1922). On the interpretation of χ2 from contingency tables, and the calculation of P. J R Stat Soc.

[64] Hudson RR (2002). Generating samples under a Wright-Fisher neutral model. Bioinformatics.

[65] Foley JE, Nieto NC, Barbet A, Foley P (2009). Antigen diversity in the parasitic bacterium *Anaplasma phagocytophilum* arises from selectively-represented, spatially clustered functional pseudogenes. PLoS One.

